# Application of machine learning models on predicting the length of hospital stay in fragility fracture patients

**DOI:** 10.1186/s12911-024-02417-2

**Published:** 2024-01-30

**Authors:** Chun-Hei Lai, Prudence Kwan-Lam Mok, Wai-Wang Chau, Sheung-Wai Law

**Affiliations:** Department of Orthopaedics and Traumatology, Chinese University of Hong Kong, Prince of Wales Hospital, Shatin, Hong Kong SAR, China

**Keywords:** Machine learning, Fragility fracture, Predictive medicine, Length of stay, Geriatric hip fracture

## Abstract

**Background:**

The rate of geriatric hip fracture in Hong Kong is increasing steadily and associated mortality in fragility fracture is high. Moreover, fragility fracture patients increase the pressure on hospital bed demand. Hence, this study aims to develop a predictive model on the length of hospital stay (LOS) of geriatric fragility fracture patients using machine learning (ML) techniques.

**Methods:**

In this study, we use the basic information, such as gender, age, residence type, etc., and medical parameters of patients, such as the modified functional ambulation classification score (MFAC), elderly mobility scale (EMS), modified Barthel index (MBI) etc, to predict whether the length of stay would exceed 21 days or not.

**Results:**

Our results are promising despite the relatively small sample size of 8000 data. We develop various models with three approaches, namely (1) regularizing gradient boosting frameworks, (2) custom-built artificial neural network and (3) Google’s Wide & Deep Learning technique. Our best results resulted from our Wide & Deep model with an accuracy of 0.79, with a precision of 0.73, with an area under the receiver operating characteristic curve (AUC-ROC) of 0.84. Feature importance analysis indicates (1) the type of hospital the patient is admitted to, (2) the mental state of the patient and (3) the length of stay at the acute hospital all have a relatively strong impact on the length of stay at palliative care.

**Conclusions:**

Applying ML techniques to improve the quality and efficiency in the healthcare sector is becoming popular in Hong Kong and around the globe, but there has not yet been research related to fragility fracture. The integration of machine learning may be useful for health-care professionals to better identify fragility fracture patients at risk of prolonged hospital stays. These findings underline the usefulness of machine learning techniques in optimizing resource allocation by identifying high risk individuals and providing appropriate management to improve treatment outcome.

## Introduction

In Hong Kong, the population of people aged 60 or above is expected to increase from 1.2 million (18% of the entire population) in 2009 to 3.4 million (39% of the entire population) in 2050 [1]. With the trend of increasing population in the elderly population, fragility fractures are becoming more common injuries due to falls and bone quality deterioration. Moreover, hip fracture, a type of fragility fracture, is now one of the most common causes of patient hospital admission, resulting in high morbidity and mortality. The annual risk of hip fracture in 2010 was 3.0 per 1000 patients in males and 6.1 per 1000 in females [2]. Patients with fragility fractures face reduced mobility and loss of independence after injury. In addition, the recovery process carries the patients through different hospitalization phases which demand a comparatively long length of hospital stay before returning to the community [3]. Hong Kong population-based analysis on the incidence of fragility fractures, characteristics, and length of hospital stay from 2004 to 2018 reported nearly half of all patients had secondary fractures in the first two years, and falls were the major cause of fractures [4].

Our previous study reported as high as 4.1% for in-hospital mortality in fragility fracture patients [5]. Another report from our group illustrated 17.3% of fragility fracture patients died within 1 year, compared with the 1.6% mortality rate in Hong Kong’s age-matched population [6]. Fragility fracture affects multiple body systems; therefore, it is associated with a high rate of associated mortality.

Reducing the pressure on hospital bed capacity is one of the key challenges for the Hospital Authority. While reducing emergency admissions is difficult to achieve, reducing the length of hospital stay can improve the rate of bed turnover [7]. Hospitals can match the demand and supply for elective and emergent admissions, intensive care unit (ICU), and interhospital transfers [8]. The application of big data analysis to achieve this goal has yet to be explored. Artificial intelligence and machine learning (ML) techniques are revolutionary in fields like speech recognition and natural language processing. Prediction of patient care pathways with machine learning can help healthcare systems better understand how variability affects patients’ throughput and outcomes. Precise prediction of in-hospital mortality, 1-year mortality, and the length of hospital stay allows proper allocation of resources to the outcome in a proactive way and matches the intensity of care according to the severity of the disease.

There have been several studies applying ML techniques to help the diagnosis and management of disease. The following paragraph summarizes five similar studies applying ML techniques in the prediction of length of stay in different medical subspecialties.

A Chinese study [9] in 2020 trained various machine learning classifiers on 100,000 records of diabetic patients with 23 attributes to predict the 30-day hospital re-admission risk. Their best performing model was a random forest classifier with an area under the curve (AUC) score of 0.670. Another Chinese study [10] utilized ML algorithms to predict the length of hospital stay after total knee arthroplasty (TKA) in 2021 and concluded that this was feasible to develop ML-based models to predict LOS for patients after receiving TKA before the surgery. Results showed that most of the hospital occupants were geriatric patients, and due to their prolonged LOS, a useful predictive model of LOS provided evidence-based guidance for discharge planning and resource requirements. The AUC of the nine models developed in this study ranged from 0.710 to 0.766, with the best model being a random forest classifier. A French study [11] used 7341 structured data to predict the prolonged length of stay using 5 machine learning techniques, including logistic regression, classification and regression trees, random forest, gradient boosting and neural networks. Their best performing model was a gradient boosting classifier with an AUC of 0.810. Their variable importance analysis showed that the type of destination of the patient after hospitalization has the strongest impact on the length of stay. A Dutch study [12] in 2022 trained eight machine learning models on 5323 unique patients with 52 different features to predict the probability of unplanned readmissions within 30 days after discharge from their urology ward. Their best performing model obtained an AUC score of 0.81 and it is a gradient boosting model with XGBoost algorithm. A recently published [13] study also trained an XGBoost algorithm on 18,195 ischemic stroke patients’ electronic medical records with 28 attributes to predict their length of stay. They identified hemiplegia aphasia, the Modified Rankin Scale (MRS), National Institute of Health Stroke Scale (NIHSS) to be the top features in predicting LOS. Their best performing model had an accuracy of 0.89 under 10-fold cross validation.

A comparative summary of the above five studies is visualized in Table 1. The five studies were conducted under different specialties and the patients they recruited were not predominantly geriatric patients unlike our study, but also patients with various attributes, such as age and co-morbidity. Before setting out to apply machine learning techniques to our database, we evaluated the feasibility of this task concerning the above five studies. We identified that our goal was similar to that of those studies in calculating the length of stay or the probability of discharge using clinic data. We also noticed certain similarities between our database and theirs, mostly in terms of the number of data features and the size of the database. Understanding that contemporary machine learning algorithms had already been applied to different clinical databases across various specialties, we were confident that we could feasibly achieve similar results with our database by applying machine learning techniques. Due to the generalizability of machine learning models, we recognize the strength of machine learning is not sensitive to specific attributes of a database, be it a geriatric patients database with orthopedics-related attributes or a database featuring patients from different age groups or dealing with different specialties. When we were finetuning our models in the later stages of our study, we also referred to the five studies, aiming to achieve similar or better results (in terms of AUC) to those studies. In short, the five studies were used as evidence to support the feasibility of our project in the early stages and as a benchmark to improve our models in the later stages.

**Table 1:** Summary of five studies using machine learning techniques

Year	ML Model with best performance	Number of data features	Number of data entries	Target variable	Cut-off value	Result (AUC)
2020	Random Forest Classifiers	23	100000	Probability of unplanned readmission	30d	0.67
2021	Random Forest Classifiers	36	1298	LOS after total knee arthroplasty	8da	0.766
2022	Gradient boosting classifier	17	7341	LOS after acute hospitalization	14d	0.81
2022	XGBoost	52	5423	Probability of unplanned readmissions	30d	0.81
2023	XGBoost	28	18195	LOS of ischemic stroke patients	7d and 14d	0.89

Applying ML techniques to improve the quality and efficiency in the healthcare sector is getting popular in Hong Kong and around the globe, but there has not yet been research related to fragility fracture. Our main goal is to develop a predictive model on the length of hospital stay (LOS) of geriatric fragility fracture patients, and a simple, reliable, and easy-to-score mortality assessment tool, named “Fragility Fracture Mortality Index (FFMI)” using artificial intelligence and ML techniques. Apart from our main objective, we also would like to validate the predictive model and FFMI by applying the model and FFMI in routine clinical practice. Besides, we aim to carry out a comprehensive summary of the epidemiology of fragility fracture in Hong Kong.

In this study, we have three major hypotheses. The predictive model can achieve a relatively high accuracy in predicting the length of hospital stay, in terms of Area Under Curve (AUC). The successful development of FFMI for fragility fracture patients can predict the likelihood of death in the hospital and within 1 year after fragility fracture in terms of percentage mortality. Based on metrics, such as patient’s demographic features, functional outcome scores and service quality control parameters, we will have a better understanding of the change of impact of patients’ medical complexity and factors causing the actual length of hospital stay.

## Methods

**Fig. 1:**
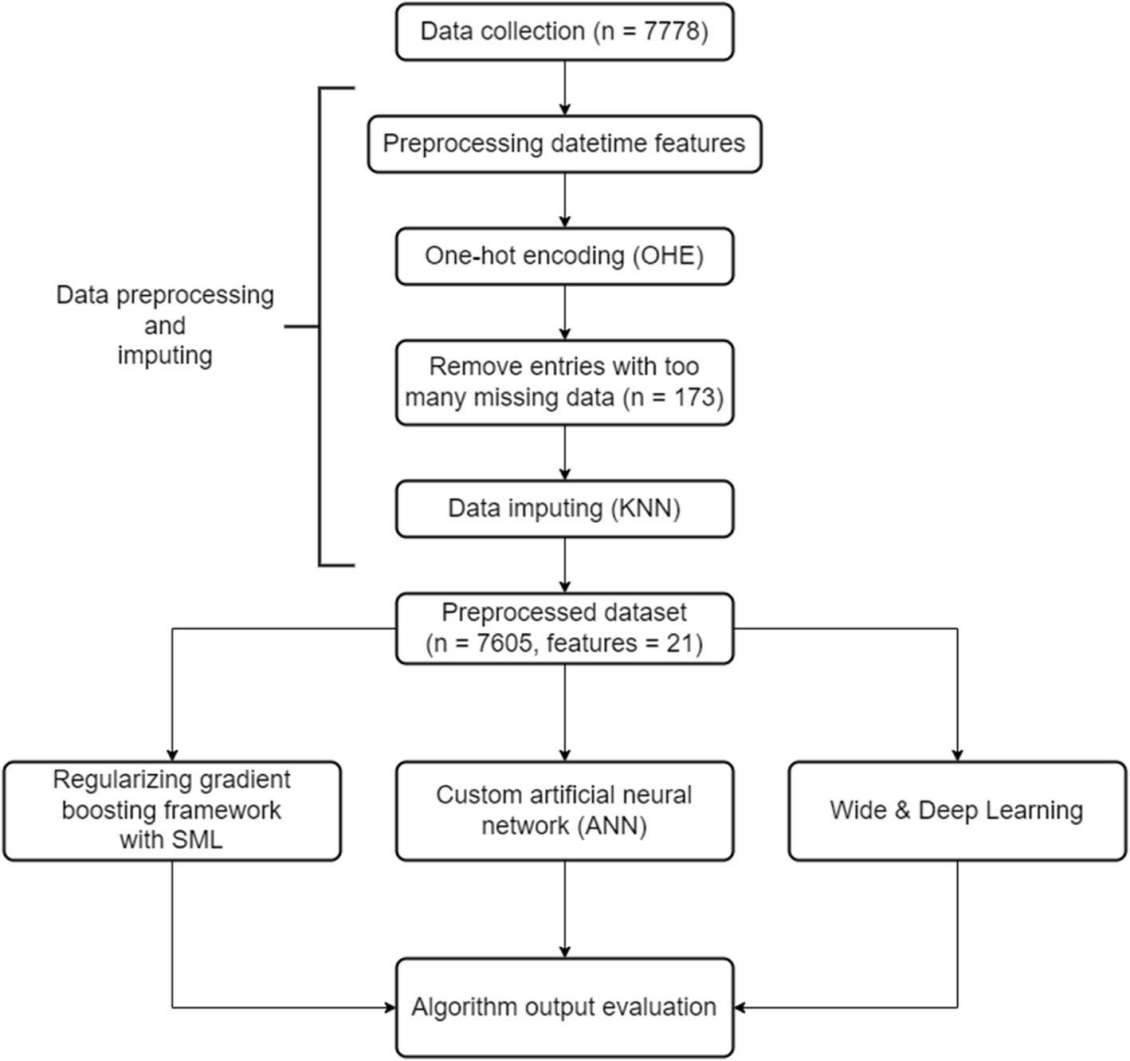
Overview flowchart for machine learning process

An overview for the whole process of our research approach can be found in Fig. 1.

### Step 1: Data collection and feature selection

All hip fracture patients aged 65 years and older discharged from Orthopaedic rehabilitation wards in Tai Po Hospital will be recruited. This study is an extension of our existing hip fracture study, which started in the year 2010. Our research assistant visits Orthopaedic rehabilitation wards in Tai Po Hospital to collect data regularly. Nurses and allied health professional colleagues help fill out a standard data collection form and the research assistant enters the data into a laptop on-site. We have already collected 7778 fragility fracture records in the said study period. Data collection will continue, and the database will keep updating and expanding with new patient records and follow-up records. Inclusion criteria were all hip fracture patients aged 65 years and older discharged from Orthopaedic rehabilitation wards, at Tai Po Hospital. Exclusion criteria were those patients discharged other than hip fracture or hip fracture patients younger than age 65.

All information was collected through electronic medical records (CMS) through the hospital electronic record system (cluster based) and the rehabilitation progress reports from the physiotherapy department and occupational therapy department in Tai Po Hospital. The basic information collected and retrieved through CMS includes: 1) Date of admission to acute hospital, 2) Date of discharge from acute hospital, 3) Date of admission to palliative hospital, 4) Date of discharge from palliative hospital, 5) Gender of the patient, and 6) Age of the patient. Apart from the basic information, we also have functional questionnaires carried out by experienced physiotherapists, occupational therapists and ward nurses, including 1) Elderly Mobility Scale (EMS), 2) Modified Functional Ambulatory Categories (MFAC), 3) Barthel Index (MBI), and 4) Mini-Mental State Examination (MMSE), which is later replaced by Montreal Cognitive Assessment 5-min protocol (MoCA5) due to licensing issues. We have done the score conversion of older data from MMSE and MoCA5 regarding to two studies done in 2018 [14, 15]. To further understand the background of each patient, we record the residency of the patient at admission and confirmed residency after discharge. The variable of the dataset and the sample characteristics of the preprocessed dataset can be found in Tables 2 and 3 respectively.

**Table 2:** Variable of the dataset

Categories	Variables
**Demographics**	Sex, age
**Clinical assessment**	Diagnosed type of fracture, Modified Functional Ambulatory Category (MFAC) at admission and before discharge, Elderly Mobility Scale (EMS) at admission and before discharge, Modified Barthel Index (MBI) at admission and before discharge, Cognitive assessment by the Mini-Mental State Examination (MMSE) or the Montreal Cognitive Assessment 5-min protocol score (MoCA5) ** of the patient during palliative care
**Characteristics of admission**	Destination from where the patient is admitted, Destination of discharge, Date of admission to acute hospital, Date of discharge from acute care to palliative care, Name of acute hospital, Name of palliative ward, Name of palliative hospital
**Operation features**	Date of surgery, Number of surgeries (if any) received

**Due to licensing issues, we changed from MMSE to MoCA5 in the middle of the study

**Table 3:** Sample characteristics of our dataset

Variables	Value	Description
**Year**	2010–2020	The year of hospital admission
**Ward**	3BL 3BR 3CL	The name of palliative ward
**Age**	65 +	The age of the admitted patient
**Sex**	0,1 (male = 0, female = 1)	The gender of the admitted patient
* Admission Date*^a^		The date admitted to palliative care
* Discharge Date*^a^		The date discharged from palliative care
**Pre-MFAC**	1–8	The modified functional ambulatory category of the patient on admission date
**Post-MFAC**	Same as above	The modified functional ambulatory category of the patient on discharge date
**Pre-EMS**	0–20	The elderly mobility scale of the patient on admission date
**Post-EMS**	Same as above	The elderly mobility scale of the patient on discharge date
**Pre-MBI**	0–20	The modified Barthel index of the patient on admission date
**Post-MBI**	Same as above	The modified Barthel index of the patient on discharge date
**Cognitive**	0–30	The Mini-Mental State Examination or the Montreal Cognitive Assessment 5-min protocol score^b^ of the patient during palliative care
**Diagnosis**	NOF, SUBTOF	The type of fracture (NOF, fracture neck of femur; subTOF, subtrochanteric fracture)
**Residence (from)**	HOME, OAH, OTHERS, ANHN	The type of residence from which the patient is admitted (HOME, from home; OAH, from old aged home; OTHER, from other sources of residence; ANHN, from Alice Ho Miu Ling Nethersole Hospital)
**Residence (to)**	HOME, OAH, OTHERS, ANHN	The type of residence to which the patient is discharged
**Admit Date**		The date admitted to acute hospital, usually from an accident
**DC Date**		The date discharged from acute hospital and admitted to palliative care
**Acute Hospital**	PWH, TWH, NDH, AHNH	The name of the hospital (PWH, Prince of Wales Hospital; TWH, Tung Wah Hospital; NDH, North District Hospital; ANHN, Alice Ho Miu Ling Nethersole Hospital)
**Date of surgery**		The date of surgery

^a^The *Admission Date* and *Discharge Date* are used to calculate the length of stay

^b^Due to licensing issues, we changed from MMSE to MoCA5 in the middle of our study

Python was chosen as the coding language in the ML process. Anaconda was employed as the Jupyter Notebook environment. Tensorflow provided GPU runtime support for GPU-optimized estimators. External libraries such as numpy, seaborne, matplotlib, pandas, sklearn, XGBoost, CatBoost and LightGBM were installed and imported.

### Step 2: Data preprocessing and imputing

Before feeding data into AI models, the dataset has to be cleaned up and preprocessed into an appropriate format. Date features such as “Acute admission date” or “First surgery date” was processed into time intervals, such as “First surgery date – Acute admission date” (Surg_1-Acute_Adm). Categorical features such as “Acute hospital” and “Diagnosis” are turned into vectors using one-hot-encoding or learned embedding.

Clinically collected data are often incomplete. It is impractical to only accept patient entries that contain all data. Thus, patient entries with more than 5 missing data were dropped, yielding us only 7605 viable data entries out of the original 7778 patients. For the rest of the missing data, K-nearest-neighbors (KNN) imputing method was employed.

In the end, preprocessed variables excluding length of stay, such as age, gender, the difference between admission and discharge MFAC, etc., were all used as the features to predict the LOS of the patient. The LOS is used as the label. The palliative LOS is then further preprocessed into 2 classes or 5 classes according to the classification task chosen. The descriptive statistics of the resulting preprocessed dataset at this point can be found in Table 4.

**Table 4:** Descriptive statistics for preprocessed dataset

Variable	Statistics		LOS ≤ 21	LOS > 21	Total	Correlation	*p*-value
**Age**	**Min**		27	41	27	-0.06795	3.00E-09
	**Max**		109	105	109		
	**Mean**		83.230	82.608	83.006		
	**SD**		8.716	7.959	8.455		
**Sex**	**Count**	**M**	1339	927	2266	-0.04065	0.00039
		**F**	3520	1819	5339		
**EMS**	**Min**		0	0	0	0.03336	0.003614
	**Max**		20	18	20		
	**Mean**		3.620	3.360	3.526		
	**SD**		3.360	2.668	3.131		
**MFAC**	**Min**		1	1	1	0.03784	0.037841
	**Max**		7	7	7		
	**Mean**		2.940	2.874	2.917		
	**SD**		1.218	1.046	1.160		
**MoCA**	**Min**		0	0	0	0.03784	5.66E-29
	**Max**		30	30	30		
	**Mean**		9.109	10.121	9.475		
	**SD**		7.109	6.665	6.969		
**Residence from**	**Count**	**Home**	3694	2504	6198	-0.2693	1.58E-126
		**OAH**	1165	242	1407		
**Residence to**	**Count**	**Home**	2762	1833	4595	-0.1653	9.22E-48
		**OAH**	2097	913	3010		
**Surg_1-Acute_Adm**	**Min**		0	0	0	-0.01226	0.284917
	**Max**		379	377	379		
	**Mean**		3.063	3.415	3.191		
	**SD**		13.291	15.395	14.088		
**Acute_LOS**	**Min**		0	0	0	-0.06293	3.95E-08
	**Max**		382	383	383		
	**Mean**		9.882	9.998	9.924		
	**SD**		7.661	11.827	9.380		
**Pall_Adm-Acute_Adm**	**Min**		0	0	0	-0.06896	1.74E-09
	**Max**		87	383	383		
	**Mean**		9.915	10.075	9.973		
	**SD**		5.597	11.893	8.427		
**Acute_DC-Surg_1**	**Min**		0	0	0	-0.08678	3.43E-14
	**Max**		375	652	652		
	**Mean**		8.224	7.713	8.040		
	**SD**		10.955	14.130	12.200		
**Pall_Adm-Surg_1**	**Min**		0	0	0	-0.09393	2.24E-16
	**Max**		375	652	652		
	**Mean**		8.337	7.773	8.134		
	**SD**		11.025	14.161	12.253		
**Pall_Adm-Acute_DC**	**Min**		0	0	0	-0.06310	3.63E-08
	**Max**		748	726	748		
	**Mean**		0.422	0.343	0.394		
	**SD**		13.035	13.940	13.368		
**Acute hospital**	**Count**	**1**	1678	1143	2821	N/A	N/A
		**2**	979	695	1674		
		**3**	313	280	593		
		**4**	244	122	366		
		**NA / Others**	2151		2151		
**Diagnosis**	**Count**	**1**	2408	1305	3713	N/A	N/A
		**2**	2284	1342	3626		
		**3**	106	70	176		
		**NA / Others**	90		90		

**Fig. 2:**
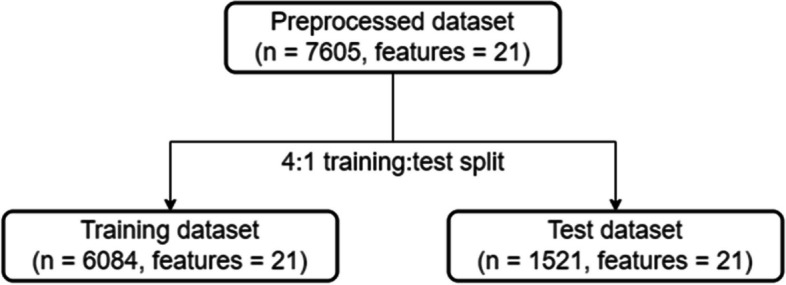
Algorithm for training-validation dataset split

After preprocessing, the whole dataset is then split into training data and testing data in a 4:1 ratio to prevent overfitting. The details of the training-test split can be found in Fig. 2.

### Step 3: Algorithm development

Depending on the decided framework and approach (SML or ANN or Wide & Deep), the training models are set up and initialized according to the specifications and hyperparameters.

For the training process, the models are first used to generate predictions, which are compared to the actual LOS values and loss are calculated for each prediction. The model would then self-calibrate and improve through normal perturbation and back-propagation. This training process was iterated to improve the model progressively until either a satisfactory result is obtained, or further training is deemed unfruitful. A satisfactory result is defined as a training model achieving sufficient predictive accuracy, with a *p*-value less than 0.05. The threshold for deeming further training unfruitful is different for each algorithm and will be discussed further below.

Satisfactory models were exported and saved for future use. Ensemble learning may be used to stack multiple satisfactory models to produce a better result. This model can be used in the future for real-time patient LOS outcome prediction or be imported into a web UI interface for user-friendly uses by doctors.

Throughout our study, we experimented with different frameworks and algorithms to explore how different algorithms perform in this scenario. The following 3 frameworks were attempted:

#### Regularizing gradient boosting frameworks with simple machine learning components

Various studies of applying machine learning techniques to calculate the length of stay at the hospital using readily available clinical data favor the usage of Classification and Regression Tree (CART) algorithms, many of them obtained favorable results with gradient boosting models, such as XGBoost algorithms [16, 17].

**Fig. 3:**
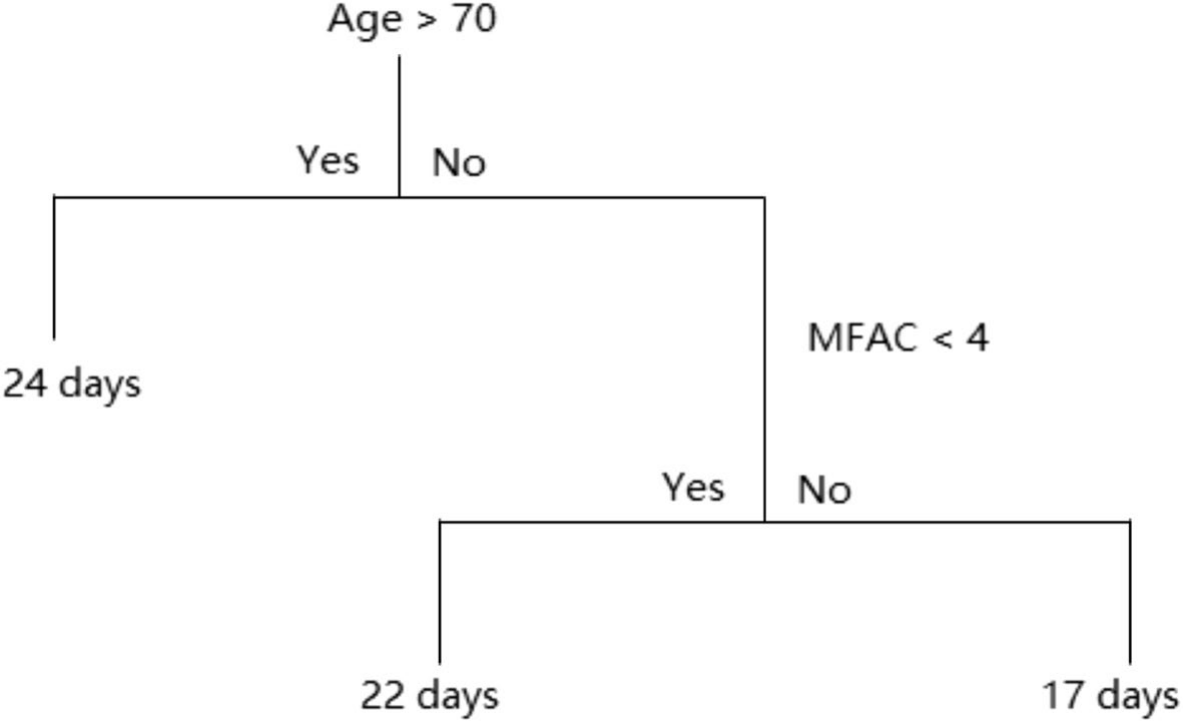
Principle of decision tree

Figure 3 demonstrates how a decision tree works with an oversimplified model. Nodes are split into sub-nodes based on a threshold value of a specific attribute, such as age being greater than 70 or not or the MFAC score smaller than 4 or not. In this simplified decision tree, if we know the patient is a 66-year-old patient with category III in MFAC, the length of stay of this patient according to this decision tree is 22 days.

Gradient boosting is also used in our algorithm It is a powerful machine-learning technique that can be used for both regression and classification tasks. It works by training a sequence of weak learners, which are usually decision trees) that are fitted on the residuals of the previous model. The final prediction is obtained by combining the predictions from all individual classifiers. However, this approach can lead to overfitting, which means that the model performs well on the training data but poorly on new, unseen data. To prevent overfitting, various regularization options are available in Gradient Boosting frameworks. Learning rates control the influence of a single learner on the final prediction, while sampling techniques select a subset of the training samples and variables to reduce complexity. For example, L1 regularization adds an L1 penalty term to the loss function, which encourages the model to have smaller weights for the features that are less important [18]. These techniques help improve the accuracy of the model by reducing overfitting and generalizing better to new data.

**Fig. 4:**
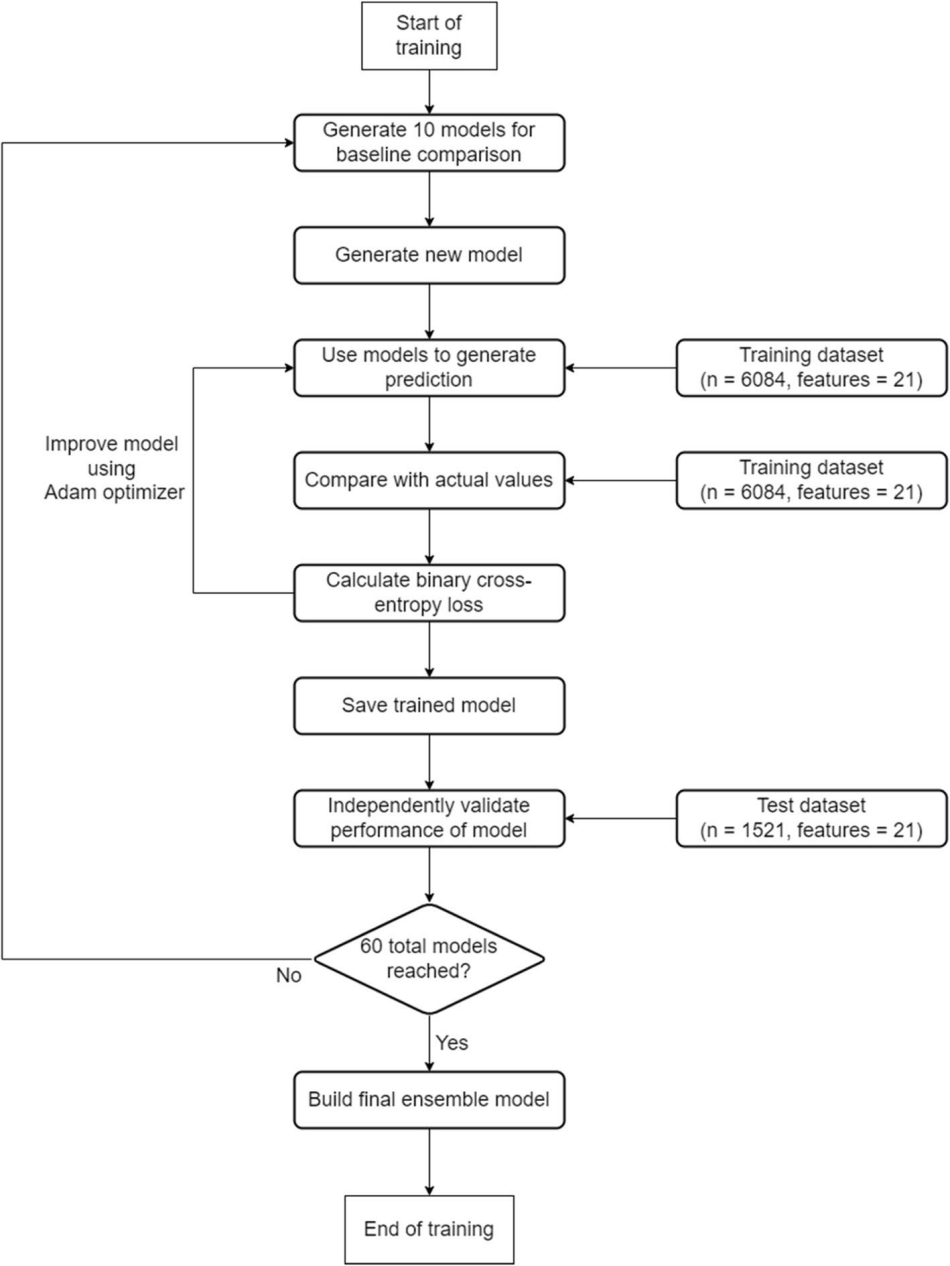
Flowchart for training regularizing gradient boosting frameworks with simple machine learning components

In our study, we experimented with various decision tree algorithms with the help of Auto-Sklearn 2.0 [19]. Auto-Sklearn 2.0 helped to train our dataset with various models, from relatively simple algorithms such as basic decision tree and random forest classfiers, to algorithms with more complexity, such as Nearest Neighbours, ExtraTrees, XGBoost, LigthGBM etc. Overview of the process for training this regularizing gradient boosting framework can be found in Fig. 4.

Log-loss function, which is also known as binary cross-entropy loss, was used as the evaluation metric for our binary classification task to predict whether the length of stay would exceed 21 days or not. The function gives a quantifiable measure, in terms of negative log-likelihood, of the difference between the predicted probabilities by the logistic model and actual values. For any given problem, a lower log loss value means better predictions. For a single sample with true label $\text{y} \in \{0,1\}$ [14], where 0 means the length of stay is smaller than 21 days and 1 means the length of stay is greater than 21 days, and a probability estimate of p = Pr(y = 1), the log loss is:

$${{\text{L}}}_{{\text{log}}}\left({\text{y}},\mathrm{ p}\right)\hspace{0.17em}=\hspace{0.17em}-\left(\mathrm{y\;log\; }\left({\text{p}}\right)\hspace{0.17em}+\hspace{0.17em}\left(1-{\text{y}}\right)\mathrm{\;log\;}\left(1-{\text{p}}\right)\right)$$

We used the log-loss function as the evaluation metric to fine-tune the hyperparameters with different models and compare the performances of different models using Auto-Sklearn 2.0. Our workflow with Auto-Sklearn 2.0 was as follows:

1. Building 10 models with basic algorithms such as decision tree, linear regression, default versions of LightGBM, XGBoost, CatBoost, Neural Network, ExtraTrees and NearestNeighbors algorithms. The ten models are used as a baseline for comparison.
2. Hyperparameters of various models, namely LightGBM, XGBoost, CatBoost, Neural Network, ExtraTrees, NearestNeighbors and Random Forest algorithms, are then finetuned for more optimal performance, using Adam as the optimizer and binary cross-entropy function as the loss function. Hyperparameters are the values that dictate the learning behaviour of the algorithm. For example, we can set the height of a decision tree or specify the learning rate of a model. Auto-Sklearn 2.0 incrementally improves the model performance by training and testing how well a model performs with specific hyperparameters.
3. After obtaining 60 models, an ensemble learning model is built based on the best performers. The ensemble model combined different algorithms, each model with different weights based on the log-loss performance, to build a final model that in theory could achieve better predictive performance than any of its constituents.
4. A leaderboard is built to reflect the performance of the models built, helping us to evaluate the performance of different algorithms on our dataset. Table 5 is an example of a leaderboard evaluating different algorithms.

**Table 5:** Example of a leaderboard of various algorithms using Auto-Sklearn 2.0

**Name**	model_type	metric_type	metric_value	train_time
[Ensemble]	Ensemble	logloss	0.610714	33.05
[1_DecisionTree]	Decision Tree	logloss	0.647846	38.1
[52_ExtraTrees]	Extra Trees	logloss	0.647803	119.35
[63_NeuralNetwork]	Neural Network	logloss	0.647378	129.13
[2_DecisionTree]	Decision Tree	logloss	0.647368	54.02
[3_DecisionTree]	Decision Tree	logloss	0.647368	60.36
[50_ExtraTrees]	Extra Trees	logloss	0.646168	92.43
[57_NeuralNetwork]	Neural Network	logloss	0.643688	58.06
[60_NeuralNetwork]	Neural Network	logloss	0.643677	94.05
[4_Linear]	Linear	logloss	0.641538	41.21
[65_NeuralNetwork]	Neural Network	logloss	0.640687	150.32
[10_Default_ExtraTrees]	Extra Trees	logloss	0.640079	59.92
[43_RandomForest]	Random Forest	logloss	0.639776	113.87
[41_RandomForest]	Random Forest	logloss	0.63973	93.73
[48_ExtraTrees]	Extra Trees	logloss	0.639579	71.18
[55_ExtraTrees]	Extra Trees	logloss	0.639313	150.11
[59_NeuralNetwork]	Neural Network	logloss	0.63795	82.64
[8_Default_NeuralNetwork]	Neural Network	logloss	0.634294	41.6
[56_ExtraTrees]	Extra Trees	logloss	0.634238	162.46
[54_ExtraTrees]	Extra Trees	logloss	0.634177	143.41
[9_Default_RandomForest]	Random Forest	logloss	0.633282	58.03
[39_RandomForest]	Random Forest	logloss	0.632654	72.66
[58_NeuralNetwork]	Neural Network	logloss	0.632352	71.92
[46_RandomForest]	Random Forest	logloss	0.631393	154.04
[62_NeuralNetwork]	Neural Network	logloss	0.631382	116.89
[61_NeuralNetwork_SelectedFeatures]	Neural Network	logloss	0.630168	170.41
[61_NeuralNetwork]	Neural Network	logloss	0.629733	106.93
[51_ExtraTrees]	Extra Trees	logloss	0.629025	108.55

#### Custom-built artificial neural network (ANN)

ANN works on the principle of biological neural networks. Each ANN composes of multiple layers and each layer composes of multiple nodes. Each node imitates a biological neuron where the input from the previous layer (imitating dendrites) is summated and the output to next layer (imitating axons) is determined by activation function (imitating axon hillocks). The nodes form a network that imitates the delicate working of brain function, and the network can gradually learn from trial and error by perturbing the weights of each input.

Multiple hyperparameters affect the performance of the ANN as well as the efficacy of the learning process. These include the width and depth of each layer, regularization, learning rate strategy, gradient descent, etc. These are changed in each run to find the optimal hyperparameters to train the best possible ANN for prediction. The hyperparameters explored and the explored values are listed in Table 6.

**Table 6:** Hyperparameters for custom ANN models

Hyperparameters	Values
Hidden layer count	{1, 2, 3, 4, 5, 6, 7}
Node count per hidden layer	{16, 32, 64, 128, 256}
Dropout layer	{true, false}
Regularizer	{None, L1, L2}
Regularization term	{0.1, 0.01, 0.001}
Learning rate schedule	{constant, linear, staircase exponential, continuous exponential}
Initial learning rate	{0.1, 0.01, 0.001}
Learning rate decay rate	{0.1, 0.25, 0.5}
Optimizer	{SGD, Adam}
Optimizer momentum	{0.8, 0.9, 0.99}

**Fig. 5:**
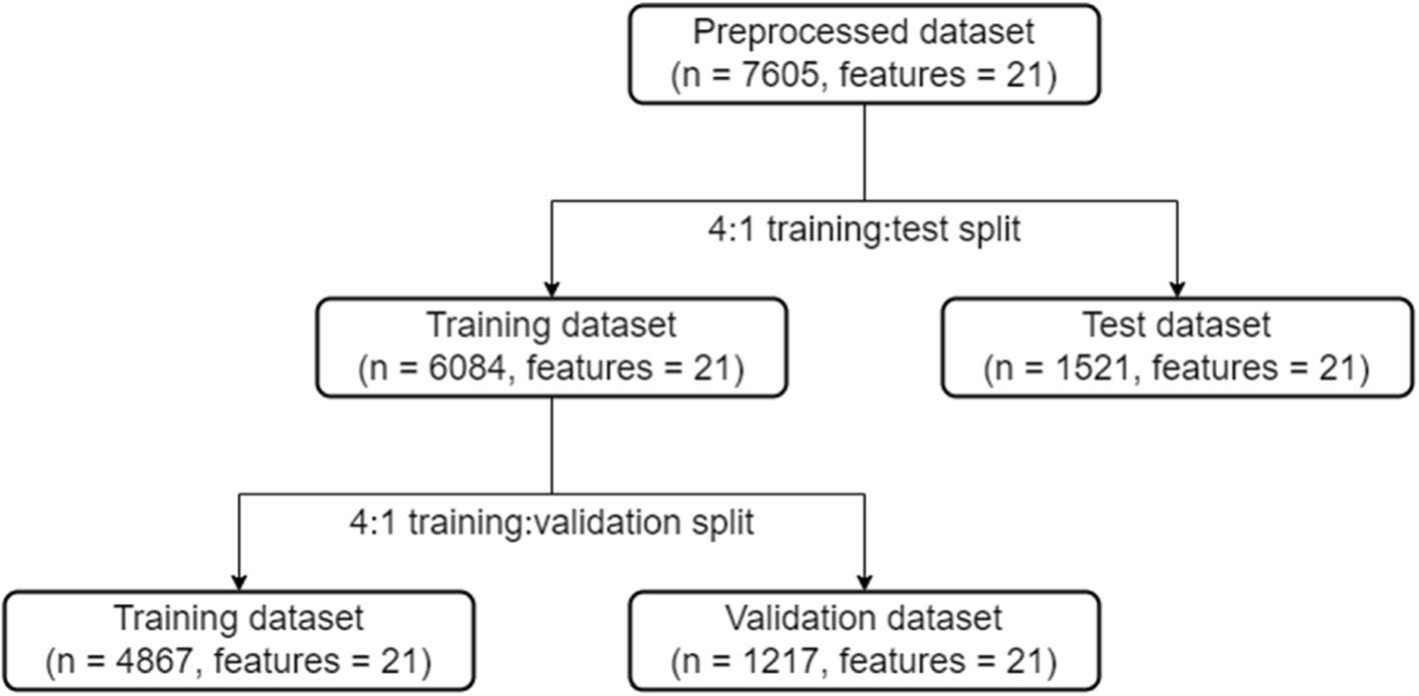
Algorithm for double 4:1 dataset split

Differing from the previous approach, other than the initial training-test split, the training dataset (*n* = 6084) is further split with a 4:1 ratio into a smaller training dataset (*n* = 4867) and a validation set (*n* = 1217). This double 4:1 dataset split is demonstrated in Fig. 5.

**Fig. 6:**
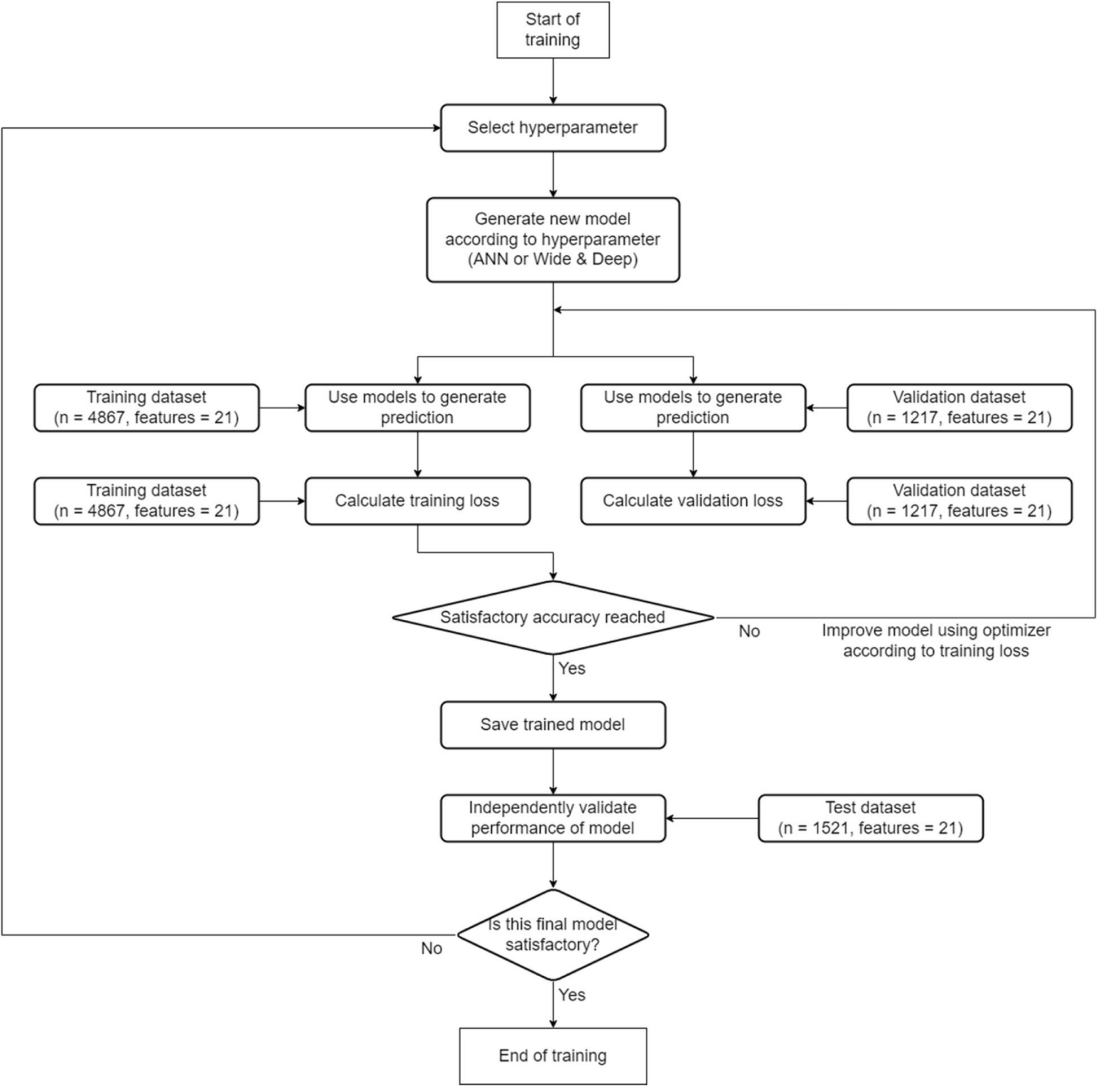
Flowchart for training custom artificial neural networks (ANN) and Deep & Wide models

During the training, hyperparameters are first chosen and an initial model is then generated according to the hyperparameters. The model is then used to generate predictions from the features of the training dataset. The generated predictions are then cross-examined with the actual value of LOS from the training dataset. Like the previous approach, binary cross-entropy is used as loss function for 2-class classification, while categorical cross-entropy is used for 5-class classification. The accuracy according to the training dataset is also calculated to track the progress throughout iterations. The loss from the training dataset is then used with the selected optimizer to update and improve the model through backpropagation and gradient descent. Then the whole process will be iterated until either a satisfactory accuracy is achieved, further iteration will be unfruitful (underfitting), or further iteration will yield worse results (overfitting). An overview of this whole ANN training process can be found in Fig. 6.

In ANN training, underfitting and overfitting are two big issues that programmers must address. During the whole process, other than the loss and accuracy generated from the training dataset, a similar process is done on the validation dataset, where predictions are made and loss and accuracy is calculated. These form 4 graphs (training_loss, training_acc, val_loss, val_acc) that help ML engineers battle underfitting and overfitting issues.

**Fig. 7:**
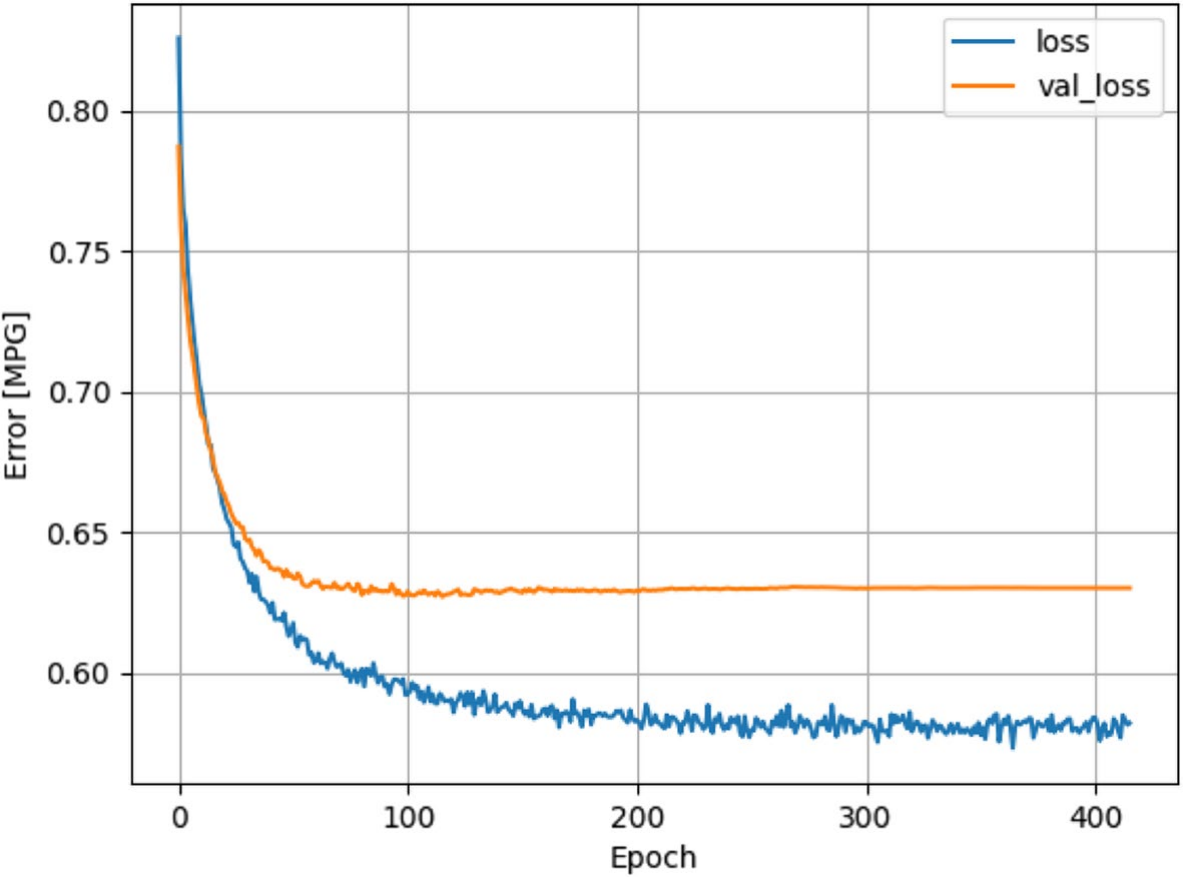
Example of binary cross-entropy loss for training and validation dataset in underfitting models

Underfitting is where the ANN model is too small that the model is unable to learn enough from the dataset and an unsatisfactory accuracy is reached. This is the easier of the two issues to spot for a ML engineer. When the loss and accuracy graphs of both training and validation dataset plateau and further progress cannot be made, this shows that this model is already trained to its best form and underfitting occurs. An example of underfitting can be found in Fig. 7. In this case, the training process will have to be halted, and hyperparameters will have to be adjusted, such as increasing hidden layer count, or increasing node count in each layer.

**Fig. 8:**
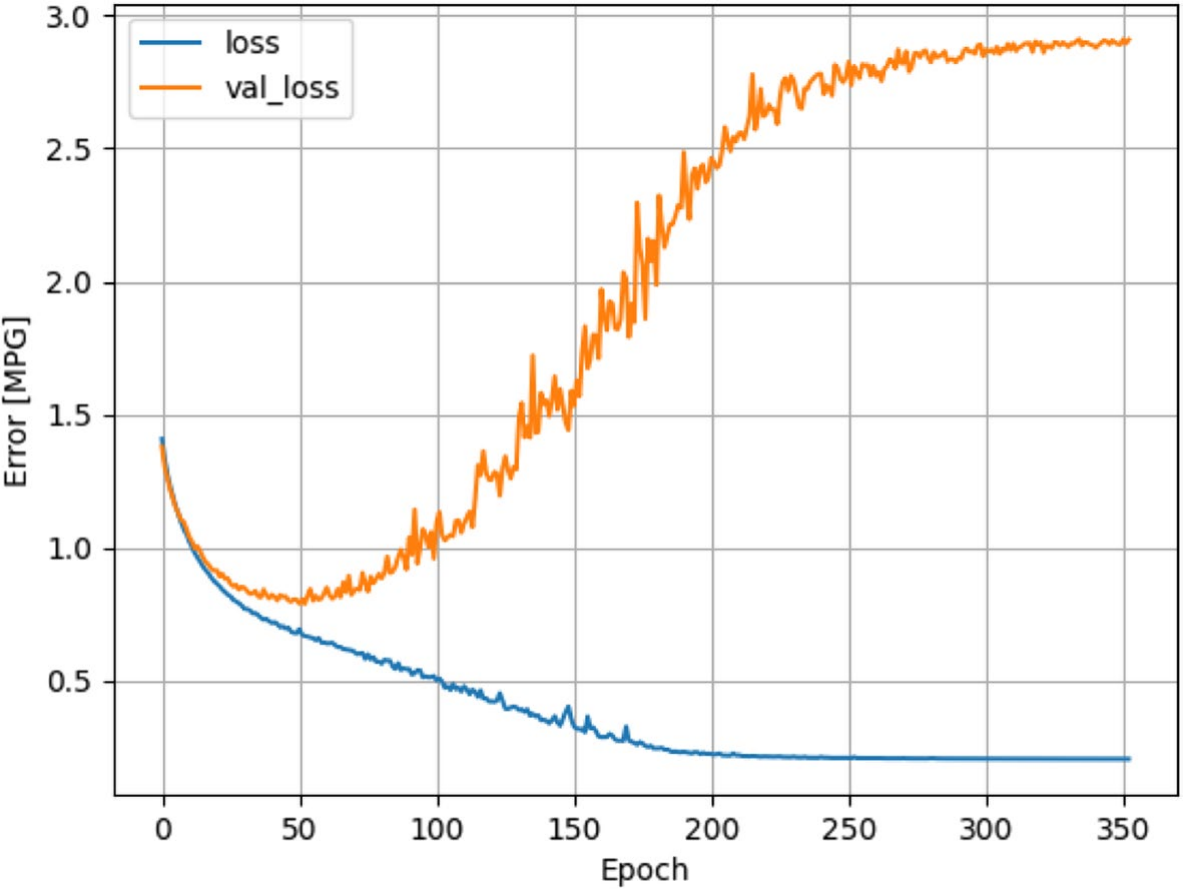
Example of binary cross-entropy loss for training and validation dataset in overfitting models

Overfitting is where the ANN model is too large with respect to the dataset. In ML training, the goal is to achieve generalization, where the model is able to learn some intricate relationships between features to make predictions. However, when the model is too large, the training process will instead achieve memorization, where the model instead just memorizes all the entries in the dataset and achieves extremely high training accuracy. This is why the initial 4:1 split generating a separate test dataset for independent assessment of model performance is important as an overfit ANN model will score a low performance with the test dataset due to lack of generalization, even though it yields high accuracy with the training dataset. For spotting overfitting, the aforementioned two graphs from the validation dataset (val_loss and val_acc) will be useful. As overfitting occurs, the model will continue to achieve progressively high training accuracy and low training loss, but the validation accuracy will start to decrease, and the validation loss will increase due to lack of generalization. An example of overfitting can be found in Fig. 8. In this case, the training process will have to be halted, and hyperparameters will have to be adjusted, such as decreasing hidden layer count, or decreasing node count in each layer. Other methods can also be employed directly in the learning process to reduce chances of overfitting, including Dropout layers, L1 regularizers or L2 regularizers.

#### Google’s Wide & Deep Learning

The approach of traditional layer by layer ANN is plagued with the problem of overfitting and underfitting. To avoid overfitting or underfitting, a fine balance between memorization and generalization is kept by keeping the ANN structure narrow and shallow.

The approach of Wide & Deep Learning proposed by Google Research combines the advantages of wide ANN and deep ANN into one [20]. With the memorization benefit of wide linear models and generalization benefit of deep models merging into one, The Wide & Deep model are able to share the benefits of both, while keeping the learning process simple. Instead of stacking layers of nodes on top of each other as in ANN, a Deep network (high depth, low width) and a Wide network (high width, low depth) are combined in the output layer with a single node with sigmoid activation.

Apart from the network structure, The whole learning process is similar to the aforementioned custom ANN model approach. An overview of this whole Wide & Deep training process can be found in Fig. 3. Similar hyperparameters are also explored in this method, including width and depth of Deep network and Wide network, regularization, learning rate strategy, gradient descent algorithm, etc. Like method 2, a large range of hyperparameter combinations are experimented with using grid search, and the best model found so far is presented below.

### Step 4: Algorithm evaluation

Model performance was determined using multiple metrics, including F1 score, R2 value and *p*-value. Model validation was addressed in the context of construct validity, reliability, responsiveness, and systematic development. With another set of data, the model was tested and validated for the accuracy of predicting (test set).

The feature importance of models is also explored using the Shapley Additive Explanations (SHAP) [21]. Feature importance analysis indicates which feature impacts the output of the ML model most.

## Results

### Demographic results

Our team has started a cohort study recruiting hip fracture patients aged 65 years and older discharged from Orthopaedic rehabilitation wards in Tai Po Hospital since the year 2010. From the year 2010 to the year 2020, the database yielded over 8000 geriatric hip fracture patients. Of these patients, 67.7% were female. The mean age was 83.6 ± 7.5 years old. 48.7% of the patients were diagnosed with a fractured neck of the femur; 48.3% were intertrochanteric hip fracture, and 2.2% were subtrochanteric hip fracture. The mean length of hospital stay was 21.3 ± 10.1 days. 79.1% lived at home before admission and 17.9% were from old age homes or hospitals. After discharge from the hospital, 56.8% returned to home while 35.5% moved to old age homes.

Allied health professionals assessed patients’ functional outcomes in terms of elderly mobility scale (EMS), modified functional ambulatory categories (MFAC), modified Barthel index (MBI), and mini-mental state examination (MMSE). EMS score at admission was 3.5 ± 2.9, and 7.9 ± 6.0 at discharge, showing a two-fold increase. MFAC score was 2.9 ± 1.1 at admission and 4.1 ± 1.6 at discharge. MBI scores were 45.6 ± 18.8 and 57.2 ± 21.7 at admission and discharge respectively.

### Predictive results of the preliminary ML models

We have developed multiple preliminary models predicting the length of hospital stay since 2019. We investigated the feasibility of using each ML framework to predict whether patients’ length of stay in a palliative hospital (LOS) is over 21 days.

**Fig. 9:**
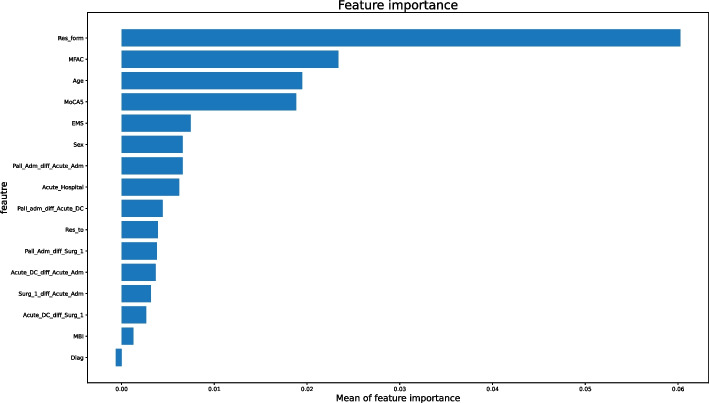
SHAP feature importance analysis of Light Gradient Boosting machine model

We developed several ML models with different frameworks to conduct this classification task using our fragility fracture cohort database. As mentioned above, we developed our ML learning models with three approaches, namely (1) regularizing gradient boosting frameworks, (2) Custom-built artificial neural network and (3)) Google’s Wide & Deep Learning,

With approach (1), we obtained the best performing model with Light Gradient Boosting algorithm, The area under the curve (AUC) was 0.73 and the F1 score was 0.68. The performance of this model can be found in Table 7. Moreover, utilizing SHAP feature importance, we found that “type of residence before admission (OAH or home)”, “MFAC”, “age”, and “MoCA5” were the four important and "impactful" factors to predict the length of hospital stay for this model. Additional information illustrating the major outcomes from this preliminary model can be found in Fig. 9.

**Table 7:** Metric details and confusion matrix of Light Gradient Boosting machine model

**Metric details**		
	**Score**	**Threshold**
logloss	0.5999256	nan
auc	0.733439	nan
F1	0.682927	0.341911
accuracy	0.678571	0.423087
precision	0.736842	0.650400
recall	1	0.061210
mcc	0.386971	0.372188
**Confusion matrix (at threshold = 0.423087)**		
	**Predicted as false**	**Predicted as true**
Labeled as false	260	143
Labeled as true	82	215

With approach (2), we also developed some models with a custom-built artificial neural network (ANN). Table 8 shows the network structure of the ANN model and its performance is listed in Table 9, yielding an accuracy score of 0.76 and an F1 score of 0.64.

**Table 8:** Network structure of our custom-built Artificial Neural Network (ANN) model

**Layer (Type)**	**Output shape**	**Param**
Normalization (Normalization)	(None,21)	43
Dense (Dense)	(None,64)	1408
Dense_1 (Dense)	(None,64)	4160
Dense_2 (Dense)	(None,64)	4160
Dense_3 (Dense)	(None,1)	65

**Table 9:** Metric details and confusion matrix of our best custom-built Artificial Neural Network (ANN) model on 2-class classification

**Metric details**		
	**Score**	
logloss	0.5796	
F1	0.6426	
accuracy	0.7653	
precision	0.7055	
recall	0.5901	
**Confusion matrix**		
	**Predicted as false**	**Predicted as true**
Labeled as false	843	134
Labeled as true	223	321

Our custom-built ANN also yielded an accuracy score of 0.47 for 5-class classification (LOS < 7, 8–14, 15–21, 22–28, > 28), as listed in Table 10.

**Table 10:** Metric details and confusion matrix of our best custom-built Artificial Neural Network (ANN) model on 5-class classification

**Metric details**					
	**Score**				
logloss	1.2933				
accuracy	0.4773				
**Confusion matrix**					
	**Predicted as LOS = 0–7**	**Predicted as LOS = 8–14**	**Predicted as LOS = 15–21**	**Predicted as LOS = 22–28**	**Predicted as LOS > 28**
Labeled as LOS = 0–7	0	12	46	4	0
Labeled as LOS = 8–14	0	131	105	9	1
Labeled as LOS = 15–21	0	85	482	68	2
Labeled as LOS = 22–28	0	30	248	104	3
Labeled as LOS > 28	0	16	92	74	9

**Fig. 10:**
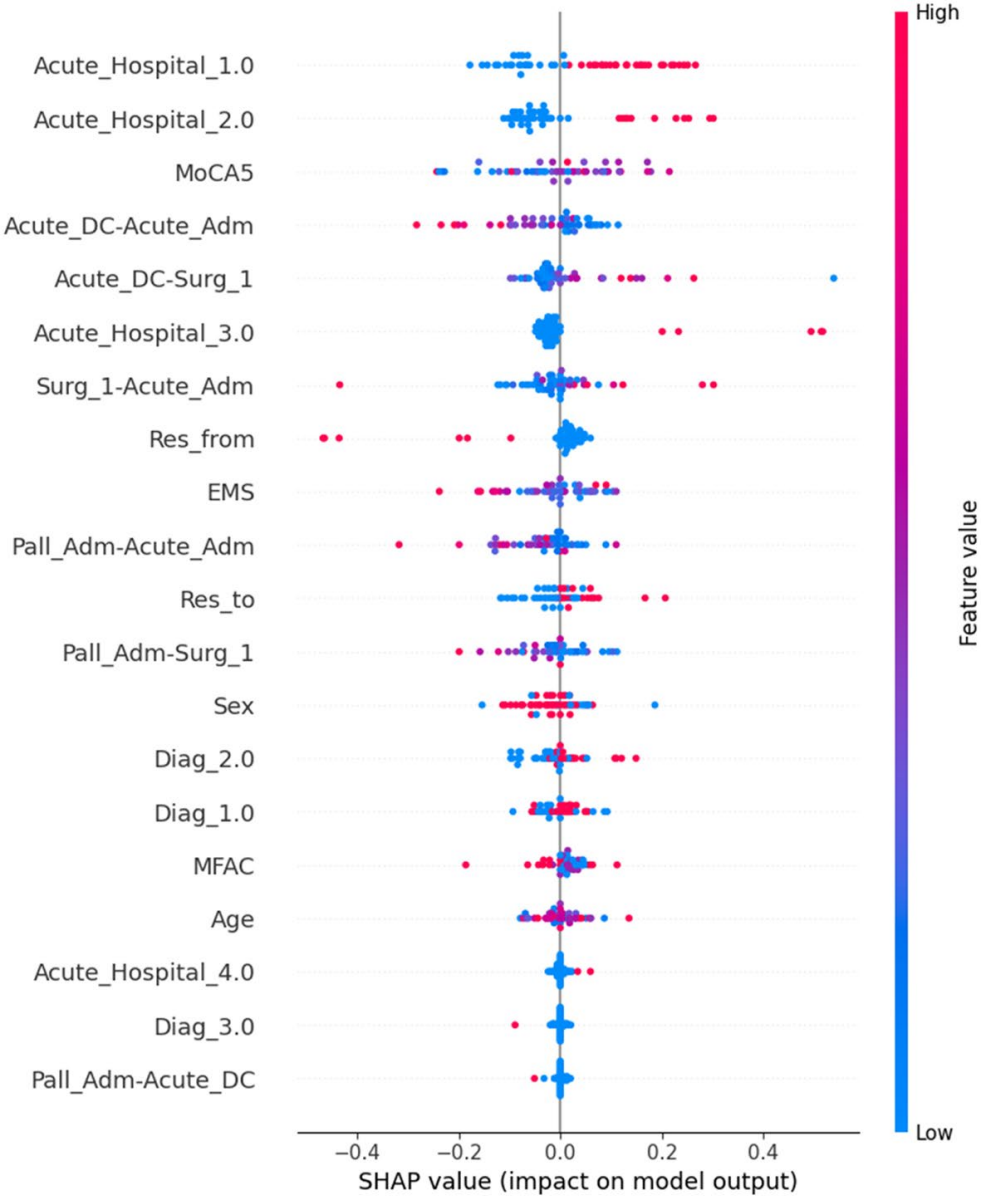
SHAP features importance analysis of our best Wide & Deep model on 2-class classification (Beehive plot)

Our best results resulted from our Wide & Deep model, which was approach (3). So far, we have achieved our best accuracy of 0.79, with a precision of 0.73, with an area under the receiver operating characteristic curve (AUC-ROC) of 0.84, as listed in Table 11. Using SHAP feature importance shown in Fig. 10, we found that “Acute_Hospital_1.0 (PWH)”, “Acute_Hospital_2.0 (TWH)”, MoCA5, “Acute_hospital_LOS” are the top 4 features of this model. This implies that the type of hospital the patient is admitted to, the mental state of the patient and the length of stay at the acute hospital all have a relatively strong impact on how long the patient would be discharged from palliative care. The comparison of the performance of the different approaches is shown in Table 12.

**Table 11:** Metric details of our best Wide & Deep model on 2-class classification

**Metric details**	
	**Score**
accuracy	0.789439
accuracy_baseline	0.655240
auc	0.843976
auc_precision_recall	0.746801
average_loss	0.489855
global_step	47600
mean	0.344759
loss	0.489683
precision	0.727354
recall	0.622648

**Table 12:** Comparison of the performance of different models

	logloss	accurary	precision	AUC
Light Gradient Boosting	0.59993	0.67857	0.73684	0.73344
ANN (2 class classifier)	0.5796	0.7653	0.7055	/
ANN (5 class cassifier)	1.2933	0.4773	/	/
Wide & Deep	0.48968	0.78944	0.72735	0.84398

Comparing our study with similar studies mentioned in our Introduction part and Table 1, our models have similar performance. This demonstrates both the increasing popularity of using machine learning techniques on readily available data obtained from electronic medical records and the relative success of the application of machine learning techniques on the prediction of clinical outcomes. Also, we observe that specialty-specific parameters help in improving the performance of model prediction outcomes.

## Discussion

This study aimed to develop a risk assessment tool to predict the LOS of geriatric hip fracture patients. Our results demonstrated that the classified physical status of the patient (MFAC score), the age, the mental status of the patient (MoCA5 or MMSE score), the type of hospital the patient is admitted to, the length of stay during acute care and the type of residence before admission were the strong predictors of prolonged LOS for palliative care.

Previous studies on risk factors leading to prolonged LOS in geriatric fragility patients had identified. Those results were consistent with most of our findings. From non-machine learning studies [22–24], researchers have identified age and classified the physical status of the patient as factors influencing LOS. In those studies, the American Society of Anesthesiologists physical status classification system was used to classify the physical status while our study used the MFAC score to categorize functional ambulation ability. Recently, a similar study [25] predicted LOS in pre-operative femoral neck fracture patients using machine learning techniques and they concluded that the age, ASA score, BMI, and time from injury to surgery were strong predictors of prolonged LOS. Their results were mostly compatible with our findings – we also discovered that age and physical status, reflected by MFAC, were strong predictors of prolonged LOS across various high-performing models.

Unique to our study, we have data attributes that are not commonly found in other geriatric fragility fracture databases. Most of the studies done on geriatric fragility fracture only have basic data features, such as gender and age [22–25], and some easily attainable data [24, 25], such as height, weight, and the International Classification of Disease (10th Revision) code, etc. Our study had more data features to more accurately reflect the situation of each holistically. Firstly, we had scores like EMS, MFAC, MBI, and MoCA5 to reflect the clinical picture more precisely for each patient. We identified the MFAC as an important factor as mentioned above, and we also noticed the mental status of the patient, reflected by MoCA5, was a strong predictor for prolonged LOS in some models. This result is consistent with a study done before [26]. Besides, we also had data to reflect the social health, for example, the type of residence before and after admission. We discovered admission from an old age home was a strong predictor of prolonged LOS in our models, suggesting the LOS is not affected by the physical health of a patient, but also the social health component of a patient – old age homes might not provide sufficient care and nutrition and not allowing adequate ambulation, and this might be the reason why our models indicated the type of admission before admission as a strong predictor of prolonged LOS.

Also unique to our study, we did not observe the relationship between surgical delay and prolonged LOS. Several previous studies [25, 27–29] have identified surgical delay as a strong predictor of prolonged LOS, although there were studies suggesting otherwise [26, 30]. In our database, our interpretation of surgical delay is defined as the date of first surgery minus the date of acute admission (‘Surg_1-Acute_Adm’), which shows the duration between the patient being admitted to the acute hospital and receiving surgery on the fracture. However, across different ML models, we did not observe ‘Surg_1-Acute_Adm’ as a strong predictor of prolonged LOS. There are several possible explanations to account for this finding. Firstly, this might be due to the inconsistency of our database – some data entries did not have the date of the first surgery leading to inaccurate calculation of surgical delay. Secondly, this might be due to the inherent inadequacy of SHAP feature importance analysis, which will be further elaborated in the following paragraphs.

Regarding the technical machine learning aspect, our study experimented with 3 types of machine learning approaches and models. Referring to similar machine learning studies [9–13] on predicted LOS in other topics under different specialties, we attainted models with similar performance. The most remarkable model, which has not been employed in other studies but has been optimized with our study, is Google’s Wide & Deep learning model, which performs better than the other two models. Like our artificial neural network models, Google’s Wide & Deep models use neural networks with loss optimization techniques to perform the supervised learning classification task. However, instead of a deep feed-forward architecture, the Deep & Wide model combines a deep feed-forward architecture for its deep component and a generalized linear model for its wide component. By doing this, it can combine the benefits of memorization using the deep component and generalization using the wide component, which easily handles the challenge of overfitting and underfitting. For data analysis and AI application in the medical field, where the goal usually focuses on generalization, yet the data are seldom linearly correlated, we recommend adding Google’s Wide & Deep learning model to the toolset for supervised learning on numerical and categorical data in medical AI research use case.

Regarding SHAP feature importance analysis, interpretation of such analysis must be cautiously made since it only indicates that the ML model regards that feature with high importance and changes in the feature’s value significantly impact the model’s output prediction. A feature having a high feature importance does not equate to having a significant statistical correlation, especially when the accuracy of the model is not significantly close to 100%. Upon doing basic statistics with Pearson’s and Spearman’s correlation, no significant correlation exists between any features and the LOS with *p* < 0.05, indicating no significant univariate correlation. In our study, we observed that we got highly different feature importance from our different frameworks, indicating the low reliability of feature importance from machine learning models with an accuracy of about 0.7–0.8 as in our study. Past empirical and theoretical studies indicate that feature importance reliability is highly correlated with model accuracy [31]. We conclude that without a high model accuracy of close to 100%, it is inappropriate to draw clinical significance and clinical decisions just from the feature importance of machine learning studies with the lack of traditional statistical correlation.

### Limitations

There is a need for additional resources to further develop our ML models to achieve predictions with higher precision.

We face several limitations in the model development process. Inconsistency in data collecting process makes the data pre-processing stage challenging. A lack of manpower in the data collection process yields a database with missing data. Our study uses assessment tools such as MFAC, MBI, etc. to evaluate the patient’s condition. However, it is extremely difficult to collect data from every single patient as both the evaluating process and the data collecting process are manpower-intensive and error-prone. Some of the values were left blank since our staff often forgot to write down the value or simply did not have time to conduct the test on the patient. The development of the ML model has thus been hampered.

Due to data privacy, the standard data collection forms cannot be taken away from the hospital premises. Research assistants must visit the Orthopaedic Rehabilitation Wards in Tai Po Hospital to collect data in person. The schedule was affected by the rapidly changing COVID-19 pandemic situation. We plan to facilitate the communication channel between us and the related staff at Tai Po Hospital by setting up regular face-to-face and Zoom meetings with different stakeholders in this project. We aim to monitor the study progress to ensure everything is on schedule.

The algorithm we are developing requires a large amount of consistent and longitudinal data. Missing data in the database would cause deviations in the algorithm results. We try to request medical records for those records with a considerable amount of missing data.

### Future work

In the future, there are several directions that we would like to embark on with our project. As mentioned in the introduction part, the mortality rate in fragility fracture is high and we would like to address this problem with ML technique as well. Our existing database already has the hospital number of every patient, and we could retrieve the mortality information to predict the chance of death based on the patients’ static features.

Inspired by the major obstacles faced in data collection, we would like to launch a web app to allow our staff to input the data directly into our database. Not only would this implementation lessen the chance of handwritten error, but this could also benefit our research assistant in data collection by not having to manually convert the handwritten forms into digital format. The web app could also provide an instant prediction of the LOS for reference.

## Conclusion

We speculate machine learning will increase the accuracy in predicting the length of hospital stay leading to better hospital resource allocation. Machine learning has a multitude of benefits to the length of hospital stay for fragility fracture patients. ML brings advantages to various stakeholders. Family members of patients can plan for the patients after discharge, e.g., arrange accommodations at old age homes, or hire a domestic helper. By identifying patients with a higher probability of lengthy LOS, doctors can allocate more resources and time to them. This can make better use of limited resources and proactively manage them to allow risk-stratified care management. Hospital administrative staff can have better resource allocation planning by learning each patient's estimated discharge destination and making data-driven decisions.

## Data Availability

The datasets used and/or analysed during the current study are available from the corresponding author upon request.
